# Monitoring Resistance of *Euschistus heros* (Fabricius) (Hemiptera: Pentatomidae) to Insecticides by Using Encapsulated Artificial Diet Bioassay

**DOI:** 10.3390/insects12070599

**Published:** 2021-07-01

**Authors:** Cristiane Maria Tibola, Leandro Silva, Fernanda Sgubin, Celso Omoto

**Affiliations:** 1 Department of Entomology and Acarology, Luiz de Queiroz College of Agriculture, University of São Paulo, Piracicaba 13418-900, SP, Brazil; celso.omoto@usp.br; 2 CL Empreendimentos Biológicos Ltd., Piracicaba 13424-586, SP, Brazil; leandro.silva@pragas.com.vc (L.S.); pesquisa@pragas.com.vc (F.S.)

**Keywords:** *Euschistus heros*, bioassay, Neotropical brown stink bug, insecticide resistance management, IRM

## Abstract

**Simple Summary:**

The Neotropical brown stink bug *Euschistus heros* (Fabricius) (Hemiptera: Pentatomidae) is currently one of the major soybean pests in Brazil, causing considerable and often irreversible damage to the crop. The main control strategy for regulating brown stink bug populations has been the use of insecticides, especially those belonging to the neonicotinoid class; however, the spraying of these insecticides does not achieve the desired control efficiency in some locations. Therefore, to improve the programs for detecting and monitoring *E. heros* resistance to insecticides, a new ingestion bioassay method by liquid diet encapsulation was evaluated in comparison with traditional bioassay methods. The new bioassay method of ingestion by encapsulation is very promising for detecting and monitoring resistance of *E. heros* populations to neonicotinoid insecticides.

**Abstract:**

The novel ingestion bioassay method was developed for detecting and monitoring resistance of *Euschistus heros* by encapsulating an artificial liquid diet using Parafilm^®^. This methodology was compared with the tarsal contact (vial test) and topical application methods for thiamethoxam, imidacloprid, and lambda-cyhalothrin. The best bioassay method for the neonicotinoid insecticides thiamethoxam and imidacloprid was ingestion. For pyrethroid insecticide lambda-cyhalothrin, the best result was obtained by topical application. Using the best bioassay method for each insecticide, the susceptibility to these insecticides was monitored in 30 populations of *E. heros* collected from soybean crops in Brazil from 2018 to 2020. High variations in susceptibility to thiamethoxam (resistance ratios, 1.6–22 times), imidacloprid (resistance ratios, 1.6–22 times), and lambda-cyhalothrin (resistance ratios, 5–40 times) were detected among the evaluated *E. heros* populations. In order to monitor the susceptibility of *E. heros* to insecticides, diagnostic concentrations were defined based on the LC_99_ of the susceptible reference population: 5.65 µL of a.i./mL for thiamethoxam, 12.45 µL of a.i./mL for imidacloprid, and 0.20 µg of a.i./insect for lambda-cyhalothrin. Subsequently, we select an *E. heros* strain resistant to neonicotinoid insecticides and another to lambda-cyhalothrin. The resistance ratios obtained after seven selection cycles were 66, 41 and 44 times for thiamethoxam, imidacloprid and lambda-cyhalothrin, respectively.

## 1. Introduction

The Neotropical brown stink bug *Euschistus heros* (Fabricius) (Hemiptera: Pentatomidae) is currently one of the major pests of soybeans (*Glycine max* (Merril)) in Brazil, causing significant and often irreversible damage to crops [1,2,3]. Brown stink bugs colonize soybean plants during the vegetative phase and are detrimental from pod formation until grain ripening. The damage caused by this species results from the insertion of the stylet in the soybean plants or pods, wherein they inject salivary secretions facilitating the feeding process. In addition, they can promote the development of fungal diseases and cause physiological disorders, such as leaf retention, compromising yields by up to 30% if they feed on pods throughout their reproductive stages [4,5,6,7,8,9]. Due to the severe damage caused, some authors acknowledge phytophagous brown stink bugs as the most important pest of soybean cultivation [10,11].

Overall pest control in soybean is practically based on the use of chemicals for insects and mites as well as for weeds and diseases affecting the crop [12,13]. It is estimated that four to eight insecticide applications are currently performed per soybean crop season [10,14,15]. Insecticide sprays for the control of phytophagous brown stink bugs have been frequently used, including insecticides belonging to three chemical groups (neonicotinoids, organophosphates, and pyrethroids), which can be used alone or in formulated mixtures [16]. The limited availability of insecticides and frequent use of the same mode of action may favor the selection of resistant populations, especially if insect resistance management strategies (IRM) are neglected [17,18,19]. The application of many of these insecticides has not achieved the desired control efficiency, and the high number of applications has not reduced the population increase of *E. heros* in different crops [10,11,14].

In Brazil, *E. heros* control measure failures have been reported for several insecticides, including beta-cyfluthrin, bifenthrin, lambda-cyhalothrin (pyrethroid), and imidacloprid (neonicotinoid) [19,20,21,22]. Until now, cases of *E. heros* resistance to endosulfan (cyclodiene), monocrotophos (organophosphate), methamidophos (organophosphate) [23,24], and imidacloprid [25] were detected using the tarsal contact method.

The methods currently used to detect and monitor brown stink bug resistance to insecticides have been based on topical and tarsal contact bioassays [23,24,26,27,28,29,30,31]. However, it is known that the detection of resistance can be affected by the chosen bioassay method [32,33,34,35], and topical and tarsal contact bioassays are based on direct contact of the insect with the insecticide, i.e., topical application to the insect integument or tarsal contact by vial test. Neonicotinoids, the main group of insecticides used to control sucking pests, have systemic properties, with physicochemical characteristics that allow their entry and translocation to all parts of plant tissues [36,37,38]. Further, neonicotinoids are rapidly degraded when the plant is exposed to UV radiation [39].

Developing resistance management strategies for *E. heros* requires a bioassay method for systemic insecticides, which can be reproduced easily for practical application, provides adequate dose−response data for statistical analysis, and enables detection of low resistance frequency levels [40]. Therefore, due to the inherent characteristics of insecticides belonging to the neonicotinoids class and increase of the *E. heros* population causing damage to soybean and other crops, the development of a new bioassay method that simulates the systemic action of insecticides and the form of suction feeding of brown stink bugs was needed. Therefore, the objective was to detect, monitor and select the resistance of *E. heros* to the insecticides thiamethoxam, imidacloprid and lambda-cyhalothrin using ingestion bioassay with encapsulated artificial diet compared to traditional methods of tarsal contact (vial test) and of topical application.

## 2. Materials and Methods

### 2.1. E. heros Populations

To evaluate the traditional bioassay methods of tarsal contact and topical application, compared with the proposed new ingestion method, a susceptible reference strain of *E. heros* (SUS) was used. This strain was originated from insects collected in soybean fields at Areião Farm at Luiz de Queiroz College of Agriculture, University of São Paulo, Piracicaba, São Paulo, Brazil. The SUS strain has been maintained for more than 6 years in the laboratory of the company Pragas.com^®^, in the absence of selection pressure from insecticides (Table 1).

**Figure 1:**
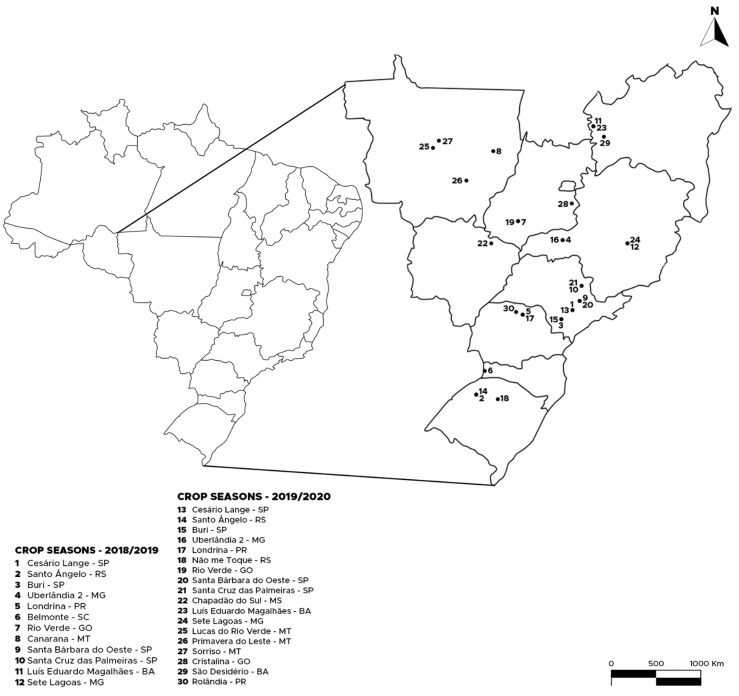
Collection sites of the populations of *E. heros* used in the bioassay for selection and characterization of susceptibility to insecticides.

Studies of susceptibility to insecticides were performed in *E. heros* populations collected in the 2018/2019 and 2019/2020 seasons from soybean crops from different regions of Brazil (Table 1; Figure 1). After collection, brown stink bugs were brought to the laboratory and were maintained in aerated plastic containers (50 cm long × 30 cm wide × 15 cm high). The brown stink bugs were fed with green bean pods (*Phaseolus vulgaris* L.), soybean seeds, and peanuts (*Arachis hypogaea* L.). The rearing of *E. heros* and the bioassays were performed in an air-conditioned room at 25 °C ± 2 °C, 60% ± 10% RH, and 12:12-h photoperiod. The insects collected in the fields were kept under the above mentioned conditions and used the F_1_ generation of each field population for bioassays.

### 2.2. Insecticides

The technical insecticides used to evaluate the susceptibility of *E. heros* populations by traditional tarsal contact and topical application methods were as follows: lambda-cyhalothrin (≥95% a.i.; Sigma-Aldrich, Saint Louis, MO, USA), which is a modulator of sodium channels (IRAC MoA 3A pyrethroid group); thiamethoxam (≥98% a.i.; Sigma-Aldrich; Saint Louis, MO, USA) and imidacloprid (≥98% a.i.; Sigma-Aldrich, Saint Louis, MO, USA), which are competitive modulators of nicotinic acetylcholine receptors (IRAC MoA 4A neonicotinoid group).

Three commercial insecticides were used to evaluate the susceptibility of *E. heros* populations by the ingestion method: lambda-cyhalothrin (IRAC MoA 3A pyrethroid group; Karate Zeon 50 CS; 50 g a.i./L in 200 L/ha spray volume; Syngenta Protecção de Cultivos Ltd.a, São Paulo, Brazil), thiamethoxam (IRAC MoA 4A neonicotinoid group; Actara 250 WG; 250 g a.i./L in 200 L/ha spray volume; Syngenta Protecção de Cultivos Ltd.a, São Paulo, Brazil), and imidacloprid (IRAC MoA 4A neonicotinoid group; Evidence 700 WG; 700 g a.i./kg in 200 L/ha spray volume; Bayer, São Paulo, Brazil).

### 2.3. Bioassay Methods

#### 2.3.1. Ingestion

The proposed new method of ingestion involved incorporation of the insecticide to be tested in an artificial diet. The artificial diet used was modified from Cerna-Mendoza et al. [41] (Table 2) to obtain a liquid diet with an even mixture of the insecticides. For diet preparation, the bean pods were washed under running water, cut, and frozen for 60 h to be lyophilized (Savant Freeze Dryer, model Novalyphe—NL150). Subsequently, the material was blended and stored in airtight containers. During diet preparation, peanuts were ground and later mixed with the other ingredients in the blender. The liquid diet was stored in a refrigerator for a maximum of 3 days.

**Figure 2:**
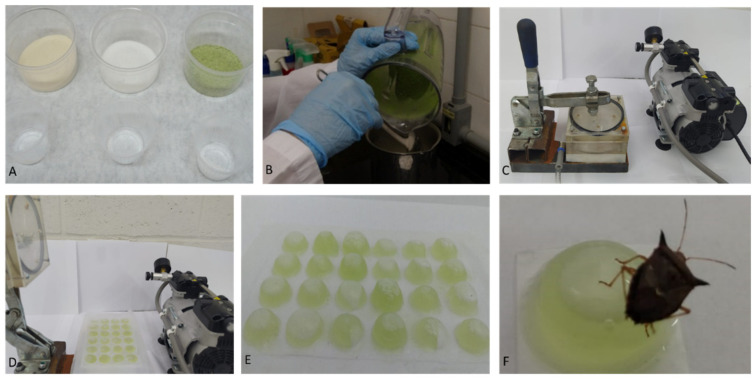
(**A**) Ingredients of the artificial diet; (**B**) mixing in a blender; (**C**) preparation of the capsules with the mold and the press coupled to the vacuum pump; (**D**) capsules containing artificial diet already sealed; (**E**) capsules containing the artificial diet; (**F**) *E. heros* feeding on the artificial diet capsule.

The proposed bioassay ingestion method was developed by the encapsulation of a liquid artificial diet, thereby enabling even incorporation of the insecticides at different concentrations, which are ingested by the insect upon feeding. The encapsulation was adapted from Greany and Carpenter [42], using an acrylic mold (13 cm length × 8 cm wide) with a press attached to the vacuum pump (Tecnal, model TE-0582). The acrylic mold containing 24 wells was coated with sealing film (Parafilm M^®^, Bemis American, Neenah, WI, USA) for forming and shaping the wells with the aid of the vacuum pump for depositing 1.0 mL of the artificial liquid diet in each well. Then, a layer of the sealing film was placed over the diet-containing wells and pressed for sealing and closing the capsules. Each capsule was 1.2 cm in diameter and 0.5 cm in depth (Figure 2).

**Figure 3:**
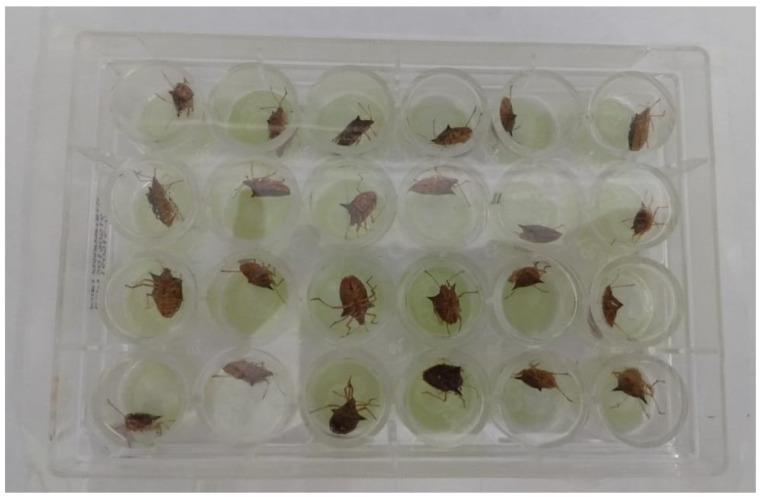
*E. heros* feeding on the artificial diet capsules containing the insecticides concentrations in 24-well plate sealed with Parafilm M^®^.

Five to nine concentrations prepared from commercially available products of each insecticide were diluted in water and added to the artificial liquid diet to be tested. Adult brown stink bugs were individualized in each cell of 24-well plates (Kasvi, model K12–024) containing one capsule of liquid artificial diet. Then, each plate was sealed with Parafilm M^®^ to avoid that one insect moving to another cell. Feeding occurs by introducing the stylet into the capsule, perforating the Parafilm M^®^ and sucking the liquid artificial diet treated with different concentration of insecticides (Figure 3).

For each concentration of the insecticides, four replicates were tested with 24 adult brown stink bugs per concentration of each insecticide (Figure 3). Assessments of insect mortality were performed 96 h after insect exposure. Adults of brown stink bugs that did not respond with vigorous movements when touched by a brush were considered dead.

#### 2.3.2. Topical Application

For the topical application bioassay, five to nine concentrations of thiamethoxam, imidacloprid, and lambda-cyhalothrin (technical insecticides) diluted in acetone (99.5% purity; Sigma-Aldrich, São Paulo, Brazil) were used; only acetone was used for the control treatment. Using a single-channel pipette, 2 µL/insect of the products were applied to the pronotum of adult brown stink bugs. For each concentration of the insecticides, four replicates were tested with 25 adult brown stink bugs per concentration of each insecticide. The brown stink bugs were separated and distributed in 100-mL plastic containers, previously labeled according to the treatment. After the application, the brown stink bugs were kept in the same plastic containers with food (bean pods) and moistened filter paper. Mortality was evaluated 48 h after application, as indicated in the IRAC method 029 [24,30,43].

#### 2.3.3. Tarsal Contact

The tarsal contact bioassay method was performed using the vial test. Five to nine concentrations of thiamethoxam, imidacloprid, and lambda-cyhalothrin (active ingredients) diluted in acetone (99.5% purity; Sigma-Aldrich, São Paulo, Brazil) were used. As indicated in IRAC method 030 [21,23,26,27,28,29,44], for 500 μL of each concentration, acetone was added into a glass bottle (20 mL) and distributed evenly throughout the internal surface of the bottle. The bottles were rotated at room temperature using a roller stirrer (Kasvi, model K45-8010) until the acetone had completely evaporated. Following this, two brown stink bugs were placed into each container and the container was closed using a veil tissue attached with an elastic band. For each concentration of the insecticides, four replicates were tested with 25 adult brown stink bugs per concentration of each insecticide. Mortality was evaluated 48 h after continuous exposition of stink bugs on treated surface using the same mortality criteria described before.

### 2.4. Monitoring Susceptibility of E. heros Populations to Insecticides

#### 2.4.1. Estimated LC_50_/LD_50_ and LC_99_/LD_99_

The susceptibility of *E. heros* populations to insecticides was evaluated in populations collected during 2018/2019 and 2019/2020 seasons from soybean crops in different regions of Brazil (Table 1). For estimating LC_50_/LD_50_ and LC_99_/LD_99_, five to nine concentrations of lambda-cyhalothrin (active ingredient) were tested using the topical application method and commercially available thiamethoxam and imidacloprid were tested using the ingestion method, as described in Section 2.3.1 and Section 2.3.2.

The baseline susceptibility of *E. heros* to the insecticides was studied in the susceptible reference (SUS) strain to identify the diagnostic concentration, based on LC_99_/LD_99_, which could be used for monitoring populations of *E. heros* collected in different regions of Brazil, according to the criteria outlined by Ffrench-Constant and Roush [40] and Roush and Miller [45].

For the control treatment, only acetone or water was used according to the method tested. For each concentration of the insecticides, four replicates were tested with 24 adult brown stink bugs per concentration of each insecticide. Insects were kept on a natural diet for one generation before being used in the bioassays. The same mortality criteria described before was used to evaluate the bioassays.

#### 2.4.2. Diagnostic Concentration

Based on the bioassay method defined for each insecticide and diagnostic concentration, based on LC_99_/LD_99_ was carried out the monitoring of brown stink bugs in populations collected during 2018/2019 and 2019/2020 seasons from soybean crops in different regions of Brazil (Table 1). For evaluating the bioassays, the ingestion bioassay method was used for thiamethoxam and imidacloprid, and the topical application method was used for lambda-cyhalothrin, as described in Section 2.3.1 and Section 2.3.2. The diagnostic concentrations used in monitoring were based on the LC_99_/LD_99_ previously identified using the baseline susceptibility of *E. heros*. Control treatment with the susceptible reference strain included six replicates of 25 brown stink bugs. The same mortality criteria described before was used to evaluate the bioassays.

### 2.5. Selection and Characterization of Resistance of E. heros to Insecticides

To obtain a population of *E. heros* resistant to the insecticides thiamethoxam, imidacloprid, and lambda-cyhalothrin, populations collected from Londrina−Paraná (PR) and Luís Eduardo Magalhães-Bahia (BA) in the 2018/2019 season were subjected to seven to eight cycles of selection in the presence of the insecticide under laboratory conditions using the technique of mass selection. These populations were selected because they showed lower mortality at diagnostic concentrations compared to other populations in susceptibility monitoring studies (item 2.4). At the beginning of the selection process, an intermediate concentration (between the LC_50_ and LC_99_) of the SUS was used. The surviving individuals from each selection cycle were retrieved and raised on a natural diet. The insecticide concentration used for the selection was increased in the fourth selection cycle to LC_99_ of the SUS: 0.20 µg of a.i./insect for lambda-cyhalothrin (topical application method) and 5.65 and 12.45 µg of a.i./mL of diet for thiamethoxam and imidacloprid, respectively (ingestion method).

Dose−response studies of *E. heros* subjected to five to eight selection cycles in the presence of thiamethoxam, imidacloprid, and lambda-cyhalothrin under laboratory conditions were performed using five to nine concentrations of each insecticide, according to the previously described experimental design and mortality criteria.

### 2.6. Statistical Analyses

Dose–response data were analyzed using Probit analysis on the Polo-PC statistical program [46] to estimate lethal concentrations, their respective confidence intervals (95% CI), and the slope. Mortality data were analyzed using the log−log complement model [47] to estimate the diagnostic concentration based on the LC_99_. The resistance ratio was estimated by dividing the LC_50_ of the field population by the LC_50_ of the SUS [48]. The survival percentage data of *E. heros* populations were transformed to arcsine (√X/100) and were subjected to analysis of variance at a significance level of α = 0.05 [49].

## 3. Results

### 3.1. Bioassay Methods

The best method for the systemic insecticides neonicotinoid thiamethoxam and imidacloprid was ingestion via encapsulated artificial diet. For the pyrethroid lambda-cyhalothrin, topical application showed better results (Table 3).

The ingestion bioassay of thiamethoxam incorporated in the artificial diet revealed a higher slope (4.99 ± 0.54) than that of topical application (3.98 ± 0.33) and tarsal contact (2.11 ± 0.13) bioassays of thiamethoxam. A high slope value provides a steeper dose−response curve, which facilitates the distinction between susceptible and resistant individuals in resistance monitoring programs. Other factors that contribute to the choice of the ingestion method for detecting and monitoring *E. heros* resistance to the systemic insecticide thiamethoxam were the small-width confidence intervals of the estimated LC_50_ and LC_90_, in addition to the adjustment of the data to the Probit model (Table 3).

A similar trend was observed for imidacloprid, i.e., the slope for the ingestion method (3.53 ± 0.26) was higher than those for the topical application (3.36 ± 0.27) and tarsal contact methods (2.29 ± 0.17). In addition, evaluation of imidacloprid using the ingestion method resulted in small-width confidence intervals of the estimated LC_50_ and LC_90_, an adequate χ^2^, adjusted more precisely to the Probit model (Table 3).

The topical application bioassay was more suitable for evaluating the susceptibility of *E. heros* to the insecticide lambda-cyhalothrin. The results of the probit analyses presented in Table 3 indicate that the slope of the dose–response curve was higher when the insects were subjected to the topical application method (3.62 ± 0.26) compared with those when the insects were subjected to the ingestion (2.56 ± 0.17) and tarsal contact methods (2.50 ± 0.17). The topical application method provided an adequate χ^2^ and adjusted more precisely to the Probit model (Table 3).

### 3.2. Definition of Diagnostic Concentrations

The LC/LD_99_ were estimated using the mortality data of the susceptible population of *E. heros,* which were considered as diagnostic concentrations for resistance monitoring programs. According to these analyses, LC/LD_99_ for each insecticide and corresponding methods, were as follows: thiamethoxam: 5.65 (CI 4.47–8.03) µL of a.i./mL of artificial diet, by ingestion method; imidacloprid: 12.45 (CI 10.03–16.47) µL of a.i./mL of artificial diet, by ingestion method; and lambda-cyhalothrin: 0.20 µg a.i./insect, by topical application method.

### 3.3. Monitoring the Susceptibility of E. heros to Insecticides

**Figure 4:**
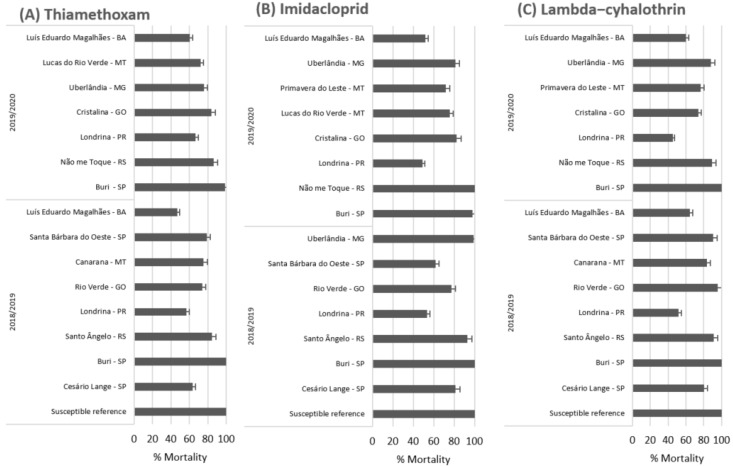
Percent mortality of populations of *E. heros* collected in crop seasons 2018/2019 and 2019/2020 exposed to a diagnostic dose of (**A**) thiamethoxam (5.65 µg of a.i./mL) in ingestion bioassays. (**B**) Imidacloprid (12.45 µg of a.i./mL) in ingestion bioassays and (**C**) lambda-cyhalothrin (0.20 µg a.i./insect) in bioassays by topical application.

High variations in the LC_50_ of thiamethoxam, imidacloprid, and lambda-cyhalothrin were observed in the populations of *E. heros* collected from different regions of Brazil between 2018 and 2020 (Table 4 and Table 5; Figure 4).

The field populations of *E. heros* showed highly variable susceptibility to lambda-cyhalothrin using the topical application method. There was no mortality in the control treatment. The LC_50_ ranged from 0.026 (susceptible population) to 1.054 (Luís Eduardo Magalhães-BA population) µg a.i./insect, representing a resistance ratio of 40 times. Other populations also showed a high resistance ratio for lambda-cyhalothrin: 20.92 times (Não me Toque-RS), 27.73 times (Londrina-PR), and 19.74 times (Primavera do Leste-MT) (Table 4). Mortality using lambda-cyhalothrin at the diagnostic concentration (LC_99_) varied among the field populations, ranging from 52.0% and 100.0% for field populations in the 2018/2019 season and between 45.3% to 100.0% in the 2019/2020 season (Figure 4).

Using the ingestion bioassay method, the concentration responses to thiamethoxam were highly variable among the tested populations. There was no mortality in the control treatment. The LC_50_ ranged between 1.79 (SUS) and 39.52 (Luís Eduardo Magalhães-BA population) μg a.i./mL with an artificial diet. The resistance ratios ranged from 1.62 to 22.08 times for the populations of Uberlândia-MG and Luís Eduardo Magalhães-BA. respectively (Table 5). When the populations of *E. heros* were exposed to the insecticide thiamethoxam at the diagnostic concentration (LC_99_), the mortality varied among the populations. This ranged from 47.2% to 100.0% in the 2018/2019 season and 54.2% to 100.0% in the 2019/2020 season (Figure 4).

For the insecticide imidacloprid, there was also no mortality in the control treatment. LC_50_ ranged from 2.68 (susceptible population) to 60.73 (population of Luís Eduardo Magalhães-BA) μg a.i./mL of the artificial diet for imidacloprid using the ingestion bioassay method. The resistance ratio ranged from 1.64 to 21.92 times for the populations of Buri−SP and Luís Eduardo Magalhães-BA, respectively (Table 5). Mortality at the diagnostic concentration (LC_99_) ranged between 45.5% and 100.0% for field populations in the 2018/2019 season, and between 49.1% and 100.0% in the 2019/2020 season (Figure 4).

### 3.4. Selection and Characterization of E. heros Resistance to Insecticides

**Figure 5:**
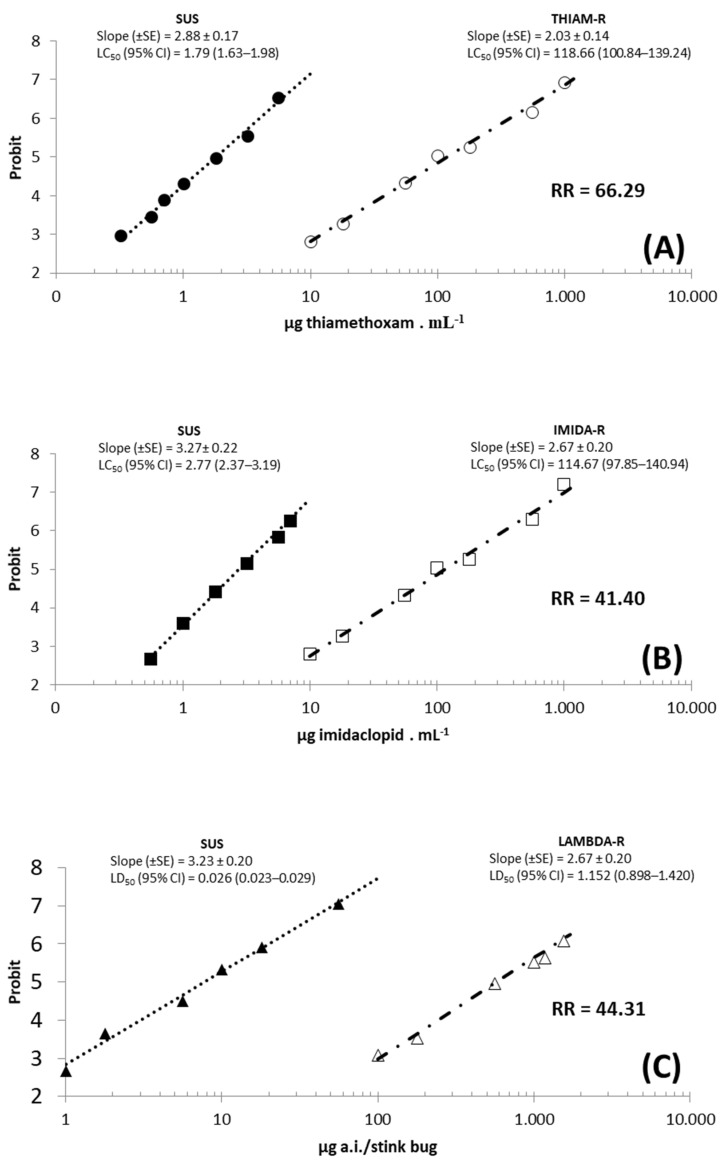
Concentration/dose-mortality responses of *E. heros* populations to the insecticides (**A**) thiamethoxam (µg of a.i./mL), (**B**) imidacloprid (µg of a.i./mL) and (**C**) lambda-cyhalothrin (µg a.i./stink bug), submitted the seven cycles of selection with insecticide in laboratory conditions, by the technique of mass selection.

In the selection and characterization of the *E. heros* population resistant to thiamethoxam (THIAM−R), the estimated population LC_50_ after eight selection cycles was 118.66 μg thiamethoxam/mL. The slope (±standard error) was 2.03 (±0.14) and the χ^2^ value was 5.33 (six degrees of freedom), with a resistance ratio of 66.29 times (Figure 5). The test of parallelism and equality of concentration−response curves estimated by the probit analysis revealed that the slope of the susceptible strain (2.88 ± 0.17) was significantly higher than that of the thiamethoxam-resistant one (2.03 ± 0.14) (Figure 5). The highest slope of the susceptible population is possibly related to the greater homogeneity of that population. Some overlapping of the concentration−response curves of thiamethoxam (THIAM−R) was present. Thus, it was not possible to determine a discriminatory concentration, but it was possible to notice an increase in the resistance ratio between the fifth and eighth selection cycles. This may be indicative of the discriminatory concentration to be used in *E. heros* monitoring programs. The diagnostic concentration was maintained at 5.65 µg of a.i./mL; this concentration caused a mortality of approximately 99% in the susceptible strain.

In the selection and characterization of the *E. heros* population resistant to imidacloprid (IMIDA−R), the estimated LC_50_ for the population after seven selection cycles was 114.67 μg imidacloprid/mL. The slope (±standard error) was 2.12 (±0.15) and the χ^2^ value was 4.66 (6 degrees of freedom), with a resistance ratio of 41.40 times (Figure 5). The test of equality and parallelism of the concentration−response curves of the resistant and susceptible populations showed significant differences in response to imidacloprid. The estimated slopes for the SUS and IMIDA−R populations were significantly different. The highest slope estimated for the SUS population was possibly related to its greater homogeneity. The concentration−response curves of the SUS and IMIDA−R populations for imidacloprid overlapped. Therefore, it was not possible to establish discriminatory concentrations, but an increase in the resistance ratio between the fifth and seventh selection cycles suggested that there was scope for further refinement in future studies on monitoring the susceptibility of *E. heros* to imidacloprid using the discriminatory concentration.

The estimated LC_50_ for the LAMBDA−R population after seven selection cycles was 1.152 µg lambda-cyhalothrin/insect. The slope (±standard error) was 2.67 (±0.20) and the χ^2^ value was 9.07 (five degrees of freedom), with a resistance ratio of 44.31 times (Figure 5). The test of equality and parallelism of the dose−response curves of the resistant and susceptible populations showed significant differences in the response to lambda-cyhalothrin. The dose−response curves of the SUS and LAMBDA−R populations for lambda-cyhalothrin overlapped. Hence, it was not possible to establish discriminatory concentrations for monitoring the susceptibility of *E. heros* to lambda-cyhalothrin.

## 4. Discussion

The new ingestion bioassay method using an encapsulated artificial diet was efficient in detecting and characterizing the resistance of *E. heros* to insecticides, as it enabled better discrimination between the susceptible and resistant populations. The proposed method of ingestion is a realistic method for systemic insecticides, especially for those belonging to the neonicotinoid class. The method is practical and can be easily reproduced. The important contributions of this method are the simulation of the systemic action of the insecticides while allowing feeding by brown stink bugs. The development of resistance management strategies requires the existence of a simple and reliable bioassay method that can provide adequate dose–response data for statistical analysis and enable the detection of low levels of resistance frequencies [50]. In addition, the capsules containing the artificial liquid diet used in the ingestion method have the thermoplastic characteristics of paraffin waxes and are flexible, odorless, moldable, malleable, translucent, colorless, easy to cut, non−toxic to insects, and easily pierced by the mouthparts of brown stink bugs.

The methods currently used to detect and monitor brown stink bug resistance are dipping bioassays of bean pods in a solution containing the insecticide, topical application bioassays, and tarsal contact bioassays using the vial test, which are based on direct contact of the insect with the product through application on the insect integument in the case of topical application or by tarsal contact of the bug in a container impregnated with the insecticide [23,24,26,27,28,29,30,31,43,44]. However, the methods of topical application and tarsal contact are not representative of the major group of insecticides used to control sucking insects. i.e., neonicotinoids. Besides, in the dipping bioassay method, which is dependent on the acquisition of bean pods, variations may arise depending on the variety, maturation stage, quality, and availability of bean pods. Therefore, realistic methods that are reproducible, regardless of the location, time of the year, and operator, are essential in integrated pest management (IPM) and IRM programs.

The resistance of *E. heros* to insecticides has not been detected using current bioassay methods, but control failures have been reported. In Brazil, failure to control *E. heros* populations has been reported for beta-cyfluthrin, bifenthrin, lambda-cyhalothrin (pyrethroid), and imidacloprid (neonicotinoid) [19,20,21,22]. Besides, there are numerous reports of farmers facing issues in controlling this species in soybean cultivation. To date, cases of *E. heros* resistance to the insecticides endosulfan (cyclodiene) and monocrotophos detected using the tarsal contact method and to the insecticide methamidophos (organophosphate) detected using the topical application method, have been reported [23,24].

One of the key objectives of resistance monitoring programs is to define a bioassay method that enables better discrimination of the susceptible and resistant pest populations [40,51]. A high slope enables maximizing the differences between the susceptible and resistant individuals; therefore, a high slope is one of the parameters for choosing the best bioassay method for evaluation [52]. In addition, a high slope allows for identifying resistance progression and genotypic variation in insecticide tolerance [53]. Therefore, high slopes obtained for thiamethoxam and imidacloprid using the ingestion method and for lambda-cyhalothrin using the topical application method indicate higher insecticidal activity and greater genotypic homogeneity of the tested populations.

The examination of the confidence intervals (95% CI) of LC_50_ for the selected bioassays, ingestion for insecticides with systemic action (thiamethoxam and imidacloprid), and topical application for insecticides with contact action (lambda-cyhalothrin) indicate that the selected bioassays are highly precise, since the 95% CI of the studied populations did not exceed twice the determined LC_50_ [54]. It was also observed that, compared with LC_50_, LC_99_ was able to better differentiate resistant individuals from susceptible individuals for the insecticides studied. Ffrench−Constant and Roush [40] reported that bioassays based on diagnostic concentrations were more efficient than those based on median lethal concentrations (LC_50_) for detecting low resistance frequencies and issues even at the initiation of resistance evolution.

Variation in susceptibility to insecticides among populations observed in distinct species of pests is not unusual [55]. From the perspective of resistance management, even a small susceptibility variation level is an indication of potential resistance selection [56]. The limited number of insecticides that can be used against brown stink bugs signifies that these insects are extensively exposed to the same active ingredients. This could result in future control failures, which can be attributed to resistance [20].

It is possible to verify an increase in the resistance ratio of some populations over the two seasons evaluated; this is likely due to the selection pressure imposed by the application of insecticides with the same active ingredient for controlling several species of insect pests throughout the crop cycle. The significant differences in the responses of the populations collected from Paraná and Bahia in the evaluated seasons indicate the possible existence of differences in the regime of insecticides used between these regions, which may be higher compared with those used in other regions of Brazil. However, the crop management and ecological situation of these locations are distinct. Londrina-PR is predominantly involved in the agriculture of soybean and maize, has a mild climate in the winter, and is warm in the summer. This causes brown stink bugs to enter dormancy in winter, which stay sheltered in straw for months and can feed on existing weeds or spontaneously grown soybean plants during off-season periods [3]. Conversely, Luís Eduardo Magalhães-BA is recognized as a hub of irrigated and high-performance agriculture of soybean and maize. Additionally, in the irrigated regions of western Bahia, brown stink bugs feed on alternative hosts since the agricultural areas succeed each other throughout the year. The number of applications of pest control chemicals is similar between the two regions, ranging from 8 to 12 applications per crop, only for agricultural pests [10,14,15]. In Londrina-PR, a commercially available mixture of lambda-cyhalothrin and thiamethoxam is most commonly used to control brown stink bugs in soybean; in contrast, in Luís Eduardo Magalhães-BA, imidacloprid, thiamethoxam, and a commercially available mixture of lambda-cyhalothrin and thiamethoxam is used to control brown stink bugs in soybean.

Our studies indicated high variation in susceptibility to insecticides in populations of *E. heros* in Brazil, as well as in responses to selection for resistance to thiamethoxam, imidacloprid, and lambda-cyhalothrin using a novel ingestion bioassay method. This reinforces that management practices are urgently needed to delay the evolution of insecticide resistance in *E. heros* populations in the field.

## 5. Conclusions

The new bioassay method of ingestion by encapsulation of an artificial liquid diet is very promising for detecting and monitoring resistance of *E. heros* populations to neonicotinoid insecticides. This data is essential for developing IPM and IRM programs that consider various brown stink bug control strategies and providing the most appropriate bioassay method and diagnostic concentrations for monitoring *E. heros* resistance to thiamethoxam, imidacloprid and lambda-cyhalothrin.

## Figures and Tables

**Table 1:** Populations of *E. heros* used in the evaluation of bioassay methods for selection and characterization of susceptibility to insecticides.

Crop Seasons	Populations	Collection Site	Latitude	Longitude	Collection Date
	SUS	Piracicaba—SP	−22.413.920	−47.382.900	January 2013
2018/2019	1	Cesário Lange—SP	−23.221.722	−47.879.901	January 2019
	2	Santo Ângelo—RS	−28.268.440	−54.222.377	February 2019
	3	Buri—SP	−23.797.690	−48.555.636	February 2019
	4	Uberlândia 2—MG	−19.039.666	−48.214.689	January 2019
	5	Londrina—PR	−23.270.385	−51.096.414	January 2019
	6	Belmonte—SC	−26.864.231	−53.579.014	February 2019
	7	Rio Verde—GO	−17.800.507	−50.888.559	January 2019
	8	Canarana—MT	−13.545.447	−52.326.032	February 2019
	9	Santa Bárbara do Oeste—SP	−22.826.796	−47.469.803	January 2019
	10	Santa Cruz das Palmeiras—SP	−21.846.312	−47.248.144	January 2019
	11	Luís Eduardo Magalhães—BA	−12.158.124	−45.781.697	January 2019
	12	Sete Lagoas—MG	−19.447.287	−44.174.600	January 2019
2019/2020	13	Cesário Lange—SP	−23.221.823	−47.882.271	December 2019
	14	Santo Ângelo—RS	−28.271.968	−54.229.174	February 2020
	15	Buri—SP	−23.797.690	−48.555.636	February 2020
	16	Uberlândia 2—MG	−18.931.495	−48.165.026	January 2020
	17	Londrina—PR	−23.246.961	−51.119.236	January 2020
	18	Não me Toque—RS	−28.450.782	−52.844.472	January 2020
	19	Rio Verde—GO	−17.854.921	−50.947.760	January 2020
	20	Santa Bárbara do Oeste—SP	−22.825.832	−47.470.452	December 2019
	21	Santa Cruz das Palmeiras—SP	−22.185.233	−47.242.628	December 2019
	22	Chapadão do Sul—MS	−18.757.929	−52.557.225	December 2019
	23	Luís Eduardo Magalhães—BA	−12.137.713	−45.808.886	January 2020
	24	Sete Lagoas—MG	−19.456.500	−44.173.400	December 2019
	25	Lucas do Rio Verde—MT	−13.035.200	−55.574.500	January 2020
	26	Primavera do Leste—MT	−15.505.000	−54.296.000	January 2020
	27	Sorriso—MT	−12.355.670	−55.446.880	January 2020
	28	Cristalina—GO	−16.934.871	−47.678.666	January 2020
	29	São Desidério—BA	−12.343.002	−44.996.204	January 2020
	30	Rolândia—PR	−23.161.728	−51.284.844	January 2020

**Table 2:** Composition of the artificial diet of *E. heros* for the ingestion bioassay.

Ingredients	Artificial Diet ^b^		Modified Artificial Diet ^c^	
Lyophilized and ground green beans	35.00	g	100.00	g
Crushed peanuts	35.00	g	35.00	g
Sucrose	5.00	g	10.00	g
Tetracycline	0.01	g	0.01	g
Sorbic acid	0.10	g	0.15	g
Ascorbic acid	-		0.30	g
Nipagin	1.00	g	1.00	g
Fatty acid	-		1.00	mL
Vitamin solution ^a^	-		5.00	mL
Water	30.00	mL	1000.00	mL

^a^ Composition of the vitamin solution: Niacinamide 1 g, Calcium Pantothenate 1 g, Thiamine 0.25 g, Riboflavin 0.5 g, Pyridoxine 0.25 g, Folic Acid 0.25 g, Biotin 0.02 mL, Vitamin B12 1 g, added to 1000 mL of distilled water. ^b^ Artificial diet of Cerna−Mendoza el al. [41]. ^c^ Artificial diet modified from Cerna−Mendoza el al. [41].

**Table 3:** *E. heros* concentration/dose−mortality responses to the insecticides thiamethoxam, imidacloprid and lambda-cyhalothrin by ingestion methods (µg of a.i./mL), topical application (µg a.i./insect) and tarsal contact (µg a.i./cm^2^).

Insecticide	Bioassay	n ^a^	Slope (±SE)	LC_50_/LD_50_ (95% CI) ^b^	LC_90_/LD_90_ (95% CI) ^b^	χ^2^(d.f.) ^c^	*p*
thiamethoxam	Ingestion	672	4.99 (±0.54)	1.93 (1.76–2.16)	3.49 (2.98–4.39)	3.19(4)	0.5271
	Topical application	700	3.98 (±0.33)	0.027 (0.02–0.03)	0.058 (0.052–0.068)	2.62(4)	0.0203
	tarsal contact	800	2.11 (±0.13)	0.196 (0.164–0.235)	0.795 (0.614–1.114)	5.05(5)	0.0004
imidacloprid	Ingestion	672	3.53 (±0.26)	2.73 (2.28–3.26)	6.30 (5.04–8.59)	4.89(4)	0.2982
	Topical application	700	3.36 (±0.27)	0.028 (0.023–0.036)	0.068 (0.050–0.115)	8.04(4)	0.0032
	tarsal contact	800	2.29 (±0.15)	0.315 (0.264–0.377)	1.138 (0.882–1.591)	5.26(5)	0.0001
lambda-cyhalothrin	Ingestion	768	2.56 (±0.17)	17.85 (14.87–21.49)	56.53 (43.92–79.33)	6.19(5)	0.0005
	Topical application	800	3.62 (±0.26)	0.046 (0.042–0.050)	0.104 (0.091–0.122)	2.42(5)	0.7878
	tarsal contact	800	2.50 (±0.17)	0.169 (0.143–0.199)	0.549 (0.436–0.745)	5.25(5)	0.0043

^a^ Number of tested. ^b^ LC_50_/LD_50_ e LC_90_/LD_90_: concentration/dose of insecticide required to kill 50% and 90% of stink bugs, respectively. ^c^ Degrees of freedom.

**Table 4:** Dose−mortality responses (LD) of lambda-cyhalothrin (0.20 µg a.i./insect) in bioassays by topical application.

Population	Crop Seasons	LD_50_ Estimated					
		n ^a^	Slope (±SE)	LD_50_ (95% CI) ^b^	χ^2^	d.f. ^c^	RR_50_ ^d^
Lambda-Cyhalothrin							
Susceptible reference	2019/2020	800	3.23 (±0.20)	0.026 (0.023–0.029)	3.56	5	-
Buri—SP		700	2.78 (±0.17)	0.135 (0.116–0.158)	6.46	5	5.12
Não me Toque—RS		800	3.90 (±0.24)	0.551 (0.488–0.620)	8.24	6	20.92
Londrina—PR		560	3.20 (±0.24)	0.731 (0.621–0.773)	2.99	5	27.73
Cristalina—GO		490	3.22 (±0.25)	0.291 (0.259–0.326)	4.83	5	11.02
Primavera do Leste—MT		560	2.89 (±0.21)	0.521 (0.445–0.611)	5.57	5	19.74
Uberlândia—MG		560	2.95 (±0.21)	0.132 (0.118–0.147)	3.76	5	5.00
Luís Eduardo Magalhães—BA		432	2.31 (±0.19)	1.054 (0.831–1.366)	4.44	4	39.98

^a^ Number of tested. ^b^ LD_50_: dose of insecticide required to kill 50% of stink bugs. ^c^ Degrees of freedom. ^d^ Resistance Ratio.

**Table 5:** Concentration−mortality responses (LC) of thiamethoxam (5.65 µg of a.i./mL) and imidacloprid (12.45 µg of a.i./mL) in ingestion bioassays.

Population	Crop Seasons	LC_50_ Estimated					
		n ^a^	Slope (±SE)	LC_50_ (95% CI) ^b^	χ^2^	d.f. ^c^	RR_50_^d^
**Thiamethoxam**							
Susceptible reference	2018/2019	864	2.88 (±0.17)	1.79 (1.63–1.98)	4.37	6	-
Cesário Lange—SP		432	2.49 (±0.23)	4.53 (3.99–5.12)	0.43	4	2.53
Buri—SP		648	2.39 (±0.16)	5.27 (4.32–6.47)	8.99	6	2.94
Santo Ângelo—RS		336	2.75 (±0.27)	4.36 (3.35–5.58)	4.01	4	2.44
Londrina—PR		768	1.68 (±0.13)	14.27 (11.51–18.57)	5.13	5	7.97
Rio Verde—GO		504	2.87 (±0.23)	3.09 (2.68–3.56)	3.20	4	1.73
Canarana—MT		504	2.69 (±0.22)	2.80 (2.40–3.24)	3.51	4	1.56
Santa Bárbara do Oeste—SP		671	2.43 (±0.17)	3.51 (3.06–4.00)	2.75	4	1.96
Luís Eduardo Magalhães—BA		384	2.03 (±0.25)	19.20 (15.51–25.37)	3.46	4	10.73
Buri—SP	2019/2020	432	2.75 (±0.22)	4.67 (4.04–5.38)	1.88	4	2.61
Não me Toque—RS		288	2.55 (±0.25)	4.15 (3.44–4.97)	1.01	4	2.32
Londrina—PR		504	1.96 (±0.14)	25.15 (18.94–33.58)	8.18	5	14.05
Cristalina—GO		420	2.69 (±0.21)	3.17 (2.72–3.67)	1.84	4	1.77
Uberlândia—MG		432	2.45 (±0.20)	2.90 (2.46–3.38)	2.69	4	1.62
Lucas do Rio Verde—MT		576	2.21 (±0.15)	3.79 (3.28–4.35)	1.80	4	2.12
Luís Eduardo Magalhães—BA		504	2.23 (±0.16)	39.52 (33.84–46.15)	3.49	5	22.08
**Imidacloprid**							
Susceptible reference	2018/2019	576	3.27 (±0.22)	2.77 (2.37–3.20)	4.92	4	-
Cesário Lange—SP		360	3.40 (±0.34)	9.67 (5.96–13.65)	7.12	4	3.49
Buri—SP		504	3.31 (±0.27)	4.59 (3.64–5.69)	5.54	4	1.66
Santo Ângelo—RS		336	2.59 (±0.25)	4.80 (3.99–5.73)	1.30	4	1.73
Londrina—PR		504	2.41 (±0.19)	15.36 (12.00–19.90)	7.80	5	5.55
Rio Verde—GO		504	2.78 (±0.23)	5.53 (4.79–6.34)	2.67	4	2.00
Canarana—MT		504	2.84 (±0.23)	5.46 (4.74–6.26)	2.88	4	1.97
Santa Bárbara do Oeste—SP		504	3.44 (±0.34)	9.54 (7.47–12.22)	7.30	4	3.44
Uberlândia—MG		648	2.49 (±0.18)	15.19 (12.66–18.58)	8.20	6	5.48
Buri—SP	2019/2020	420	3.10 (±0.25)	4.55 (3.97–5.19)	1.99	4	1.64
Não me Toque—RS		288	2.62 (±0.25)	4.73 (3.94–5.64)	1.35	4	1.71
Londrina—PR		504	2.32 (±0.17)	28.97 (22.51–37.09)	7.51	5	10.46
Cristalina—GO		432	2.69 (±0.22)	5.43 (4.69–6.25)	0.99	4	1.96
Lucas do Rio Verde—MT		420	2.66 (±0.21)	5.27 (4.56–6.08)	0.84	4	1.90
Primavera do Leste—MT		504	3.05 (±0.25)	20.41 (16.36–25.80)	7.94	5	7.37
Uberlândia—MG		600	2.68 (±0.19)	17.62 (13.67− 23.13)	8.78	4	6.36
Luís Eduardo Magalhães—BA		432	2.49 (±0.20)	60.73 (52.27–70.39)	2.35	4	21.92

^a^ Number of tested. ^b^ LC_50_: concentration of insecticide required to kill 50% of stink bugs. ^c^ Degrees of freedom. ^d^ Resistance Ratio.
